# 

**Published:** 1905-12-15

**Authors:** 


### CONTENTS.

| | | |
|---|---|---|
| Event and Comment | | 595 |
| Amalgam as a Filling Material, | By George Zederbaum, D.D.S. | 599 |
| Porcelain as a Filling Material, | By Henry C. Raymond, D.D.S. | 604 |
| Gold as a Filling Material, | By J. L. Sweetnam, D.D.S. | 609 |
| Discussion of Papers on Filling Materials | | 613 |
| Indents | | 627 |
| Announcements | | 635 |

